# Microbial community structure of plant-based meat alternatives

**DOI:** 10.1038/s41538-024-00269-8

**Published:** 2024-05-13

**Authors:** Franz-Ferdinand Roch, Monika Dzieciol, Narciso M. Quijada, Lauren V. Alteio, Patrick-Julian Mester, Evelyne Selberherr

**Affiliations:** 1 Centre for Food Science and Veterinary Public Health, Clincal Department for Farm Animals and Food System Science, University of Veterinary Medicine, 1210 Vienna, Austria; 2 Department of Microbiology and Genetics, Institute for Agribiotechnology Research (CIALE), University of Salamanca, 37185 Villamayor (Salamanca), Spain; 3 Austrian Competence Centre for Feed and Food Quality, Safety and Innovation FFoQSI GmbH, 3430 Tulln, Austria

**Keywords:** Microbiome, Food microbiology

## Abstract

A reduction in animal-based diets has driven market demand for alternative meat products, currently raising a new generation of plant-based meat alternatives (PBMAs). It remains unclear whether these substitutes are a short-lived trend or become established in the long term. Over the last few years, the trend of increasing sales and diversifying product range has continued, but publication activities in this field are currently limited mainly to market research and food technology topics. As their popularity increases, questions emerge about the safety and nutritional risks of these novel products. Even though all the examined products must be heated before consumption, consumers lack experience with this type of product and thus further research into product safety, is desirable. To consider these issues, we examined 32 PBMAs from Austrian supermarkets. Based on 16S rRNA gene amplicon sequencing, the majority of the products were dominated by lactic acid bacteria (either *Leuconostoc* or *Latilactobacillus*), and generally had low alpha diversity. Pseudomonadota (like *Pseudomonas* and *Shewanella*) dominated the other part of the products. In addition to LABs, a high diversity of different *Bacillus*, but also some *Enterobacteriaceae* and potentially pathogenic species were isolated with the culturing approach. We assume that especially the dominance of heterofermentative LABs has high relevance for the product stability and quality with the potential to increase shelf life of the products. The number of isolated *Enterobacteriaceae* and potential pathogens were low, but they still demonstrated that these products are suitable for their presence.

## Introduction

For most of the populations in industrialized countries, meat consumption is an integral part of the diet. In 2020, the average U.S. American and European Union citizens consumed 102 and 69 kg of meat per capita, respectively^1,2^. The global meat consumption increased from 24 kg per year and capita in 1990 to 34 kg in 2020^2,3^. Although the OECD (Organisation for Economic Co-operation and Development) estimates that consumption will level off at around 35 kg per year and capita by 2030, the total meat consumption will further increase with population growth^3^. This globally growing meat consumption plays a major role in the ecological issues we currently face, including land degradation, climate change, water pollution and loss of biodiversity^4,5^. Additionally, common industrial animal husbandry practices influence public health by facilitating the spread of antimicrobial resistances and vector-borne diseases^5–7^. Transition to plant-based diets has been identified as an effective way to tackle some of these food system’s challenges^8^. Food production needs to become a net carbon sink from 2040 onwards to reach the goal of global negative emissions^9^. The Paris Agreement predicted that the most needed changes include at least doubling the consumption of plant-based foods, including fruits, vegetables, legumes and nuts, and halving the consumption of red meat^10^. Consumers’ acceptance of the transition to a more plant-based diet is steadily growing^11,12^, and health benefits, including a lowered risk of type 2 diabetes, cardiovascular diseases and metabolic syndrome, are widely recognized^13^. In a 2021 survey conducted in ten different European countries, 2% of the participants referred to themselves as vegans, 5% as vegetarians, 3% as pescetarians and 30% as flexitarians^14^. The last group’s eating habits are focused on plant foods with the occasional inclusion of meat products. As the main target group, flexitarians account for about 90% of the sales of plant-based meat alternatives (PBMAs)^15^. Market research conducted as part of “The Smart Protein Project” noted a sales value increase of 82% for plant-based meat (vegan and vegetarian) between 2018 and 2020 for Austria^16^. The sales of this product group and the number of different products have increased strongly over the last few years^16,17^. Similar trends are recognized in other countries of the European Union^16^. Given the surging popularity of PBMAs, it is imperative to acknowledge the inherent product-specific hazards associated with their consumption. These hazards vary with the composition of ingredients, and encompass a range of contaminants including pesticides, mycotoxins, toxicologically significant plant compounds, heavy metals, and mineral oil hydrocarbons^18–20^. Despite the potential risks posed by these contaminants, research on this subject has been relatively limited. However, there is a shifting trend, with an increasing number of studies now addressing these concerns. Just recently, Mihalache et al. demonstrated the presence of significant levels of mycotoxins in the majority of the plant-based meat alternatives they analyzed. Although legal limits exist for these contaminants, they are deemed inadequate as they fail to encompass all pertinent mycotoxins and do not adequately consider current consumer behaviors^21^. Furthermore, the microbiological properties of these products have been similarly little studied to date, resulting in a corresponding lack of detailed data. This gap is particularly notable for PBMAs not classified as ready-to-eat foods, for which specific legal standards or microbiological safety criteria have yet to be established. This scenario underscores the need for enhanced research efforts to elucidate the microbiological risk profile of PBMAs, thereby informing the development of targeted safety regulations and recommendations, but also for addressing sustainability questions. Since two of the UN’s sustainability goals (goal 2 – zero hunger and 12 – responsible consumption and production) affect our eating habits, increased attention should be paid to reducing food loss and waste. A solid concept for preventing food loss and waste conveys multiple benefits: it saves food for human consumption, brings savings for primary producers, companies and consumers, as well as lowering the environmental and climate impact of food production and consumption. As 30% of food products in primary processing do not even reach the consumer, mainly because of microbial spoilage or pathogen contamination, it is essential to improve the knowledge of the microbial communities of our food, which could help to increase the shelf life, reduce the contamination with pathogens, but also to optimize packaging conditions which should be tailored for the suppression of product-specific microbiota. Still largely unanswered is the question if and how a high microbial diversity in food (consisting of living and dead microbiota) can have a positive effect on consumer’s health. It was also supposed recently that the loss of microbial diversity including the disappearance of ancestral indigenous microbiota, which is currently happening in western countries, affects human health and contributes to post-modern conditions such as obesity and asthma^22,23^. For all these research questions, fundamental knowledge on the microbial compositions on food are necessary, but still lacking. With limited knowledge regarding the microbiological properties of these products, we collected a variety of PBMAs from Austrian supermarkets to investigate their overall microbial community compositions. Additionally, we aimed to characterize the microbial profiles and compare four distinct groups of the most prevalent product types (pea and soybean based products with either “minced” (minced meat, burgers, etc.) or “fibrous” (meat chunks, fillet-like, etc.) texture). We hypothesized that products within one group have more similar communities than between groups, as a result of similar protein processing.

## Results

### Product descriptions

In total, 32 PBMA products from four groups (pea or soybean-based and minced or fibrous) were collected and summarized in Table 1. Beside these four main properties, the products were quite diverse in terms of composition and handling procedures. Particularly noteworthy was the number of ingredients used per product, ranging from 4 to 27 ingredients per product, resulting in a total of about 120 different ingredients overall (Table 1). Additionally, there was a wide range of shelf life, spanning from products with an expiry date to be consumed within a few days, to products with a best-before date, offering a shelf life of several months. The products were also different on factors that might influence the bacterial composition such as pre-heating or freezing steps or packing in modified atmosphere (MAP). Most of the products had clear cooking instructions on the labels, including the recommendation for thorough cooking (Table 1). In total, 27 samples were packed under modified atmosphere with unknown composition. Thirty samples were sold refrigerated, the other two were frozen. Additionally, according to the label, 7 of the 30 refrigerated products were frozen at any time point during the retail chain (Table 1).

**Table 1:** Showing the labelled attributes of the examined products

ID	manufacturer	shelf life^1^	cooking time^2^	no. of ingredients	additional labelling
*pea protein*^3^ *with fibrous texture*^4^					
D2	M02	7 d to bbd (1 d)	-	12	MAP
D4	M02	28 d to bbd (1 d)	3–5 min	11	MAP
D6	M02	14 d to bbd (1 d)	3–5 min	13	MAP
D7	M02	12 d to bbd (1 d)	3–5 min	13	MAP
D8	M02	28 d to bbd (1 d)	3–5 min	10	MAP
D3	M09	7 d to bbd (1 d)	-	16	MAP
*pea protein*^3^ *with minced texture*^4^					
A1	M01	9 d to ed (0 d)	7–8 min	17	consume only thoroughly heated; MAP
A4	M01	2 d to ed (0 d)	5–8 min	18	consume only thoroughly heated; MAP
A2	M02	6 d to bbd (1 d)	3–5 min	14	raw; consume only thoroughly heated; MAP
A3	M02	3 d to bbd (0 d)	4–6 min	13	MAP
A5	M02	14 d to bbd (1 d)	-	13	MAP
A7	M02	13 d to bbd (1 d)	2 min	14	MAP
A8	M02	6 d to bbd (1 d)	3–5 min	15	consume only thoroughly heated; MAP
D5	M02	12 d to bbd (1 d)	-	14	pre-heated; MAP
A6	M03	29 d to bbd (2 d)	3–5 min	6	
D1	M09	10 d to bbd (1 d)	-	16	MAP
*soybean protein*^3^ *with fibrous texture*^4^					
C1	M05	13 d to ed (-)	5 min	6	pre-heated; frozen once; MAP
C3	M05	10 d to ed (-)	4–7 min	16	pre-heated; frozen once; MAP
C4	M05	24 d to ed (-)	4–7 min	18	pre-heated; frozen once; MAP
B4^5^	M06	174 d to bbd (2 d)	6–8 min	4	consume only thoroughly heated; frozen once;
B5^5^	M06	119 d to bbd (2 d)	6–8 min	27	consume only thoroughly heated; frozen once;
C5	M07	0 d to bbd (-)	4–5 min	14	pre-heated; consume only thoroughly heated; MAP
C6	M07	0 d to bbd (-)	4–5 min	19	pre-heated; consume only thoroughly heated; MAP
C7	M07	0 d to bbd (-)	4–5 min	21	pre-heated; consume only thoroughly heated; MAP
C8	M08	8 d to bbd (1 d)	5 min	23	consume only thoroughly heated; MAP
*soybean protein*^3^ *with minced texture*^4^					
B8	M03	22 d to bbd (2 d)	3–5 min	8	
B1	M04	52 d to bbd (3 d)	4–6 min	10	MAP
B3	M04	10 d to bbd (3 d)	4–6 min	20	MAP
B2	M05	5 d to ed (-)	7 min	15	raw; consume only thoroughly heated; frozen once; MAP
B6	M05	3 d to ed (-)	8–10 min	12	raw; consume only thoroughly heated; frozen once; MAP
B7	M05	3 d to ed (-)	12 min	21	raw; consume only thoroughly heated; frozen once; MAP
C2	M05	18 d to ed (-)	5–7 min	22	pre-heated; frozen once; MAP

^1^days to expiration date (ed) or best before date (bbd) at sampling. In brackets: consume within x days after opening.

^2^Recommended cooking time. If label said (e.g.) 2 min per side, the recommended cooking time were doubled to 4 min for this table.

^3^Protein basis of the examined product. Only pea or soybean protein products were selected for the study.

^4^product designation. Products with a minced ’meat’ basis (i.e. minced meat, burger, cevapcici, sausages) were additionally classified as ’minced’, products imitating pieces of meat or a meat structure (i.e. fillets, steaks, chunks, kebab) were classified as ’fibrous’.

^5^products were sold frozen.

### Cultivable microbial communities in PBMA products

**Fig. 1:**
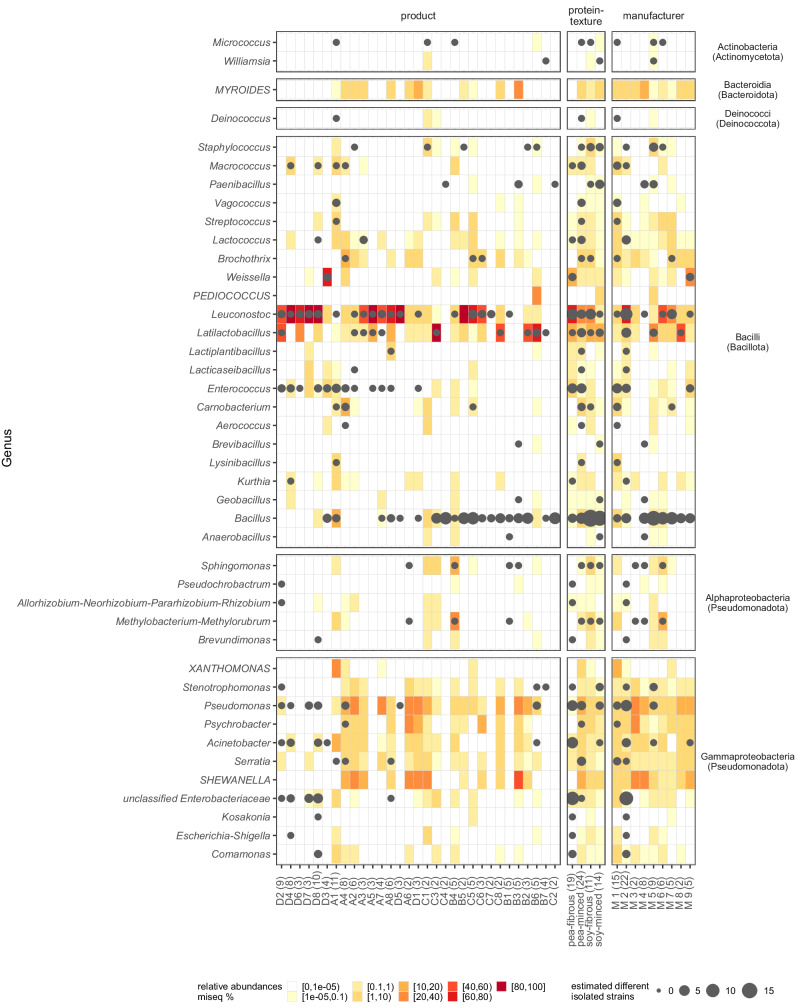
Present isolates per sample, group or manufacturer are represented by dots. The 16S rRNA gene sequences of isolates belonging to a particular genus were aligned to each other, and were clustered based on their sequence similarity. Consequently, different clusters represents different strains or species. The higher the number of clusters within a genus, the larger the plotted dot in the figure. The surrounding area is shaded according to the relative abundances in the 16S rRNA gene amplicon high-throughput sequencing (for the group and manufacturer summary, the mean relative abundance of each included sample is used). Additionally, genera, that were highly abundant in some samples, but had no corresponding isolates, were added to the figure denoted capitalized letters.

In total, 470 colonies were picked and selected for 16S rRNA gene or ITS2 region (ITS3/ITS4) Sanger sequencing. Among these, 447 (95.1%) were classified as bacteria. Of those isolates 431 (91.7% overall) were successfully classified to genus level, representing 38 genera from four different phyla (Fig. 1). Isolates classified as *Enterobacteriaceae* (*n* = 16, 3.4%) failed to reveal taxonomic classification below family level. The twenty fungal isolates (4.3% of all isolates) were classified into five different genera: *Wickerhamomyces* (*n* = 7), *Pichia* (*n* = 6), *Yarrowia* (*n* = 3), *Kurtzmaniella* (*n* = 3), *Geotrichum* (*n* = 1). Three isolates failed to retrieve any taxonomic assignment. In the initial cultivation trial, we successfully isolated most of the targeted genera. Subsequently, in the second round of cultivation, we were able to isolate *Brochothrix*, *Weissella*, and *Psychrobacter*. Despite their high relative abundances in the 16S rRNA amplicon sequencing data, *Myroides*, *Pediococcus*, *Xanthomonas*, and *Shewanella* remained elusive and could not be isolated from any of the samples. We found *Bacillus*, *Leuconostoc*, *Enterococcus* and *Latilactobacillus* very prevalent in PBMA samples, as they were isolated from 19 (59.4% from the total number of samples), 18 (56.3%), 12 (37.5%) and 10 (31.3%) samples, respectively. Moreover, species from *Bacillus*, *Leuconostoc* and *Latilactobacillus* were isolated from each of the four sample groups. *Enterobacteriaceae*, which are usually surveyed as an additional hygiene criterion, were only found in the pea protein products of a single manufacturer (Fig. 1). A selection of these *Enterobacteriaceae*, of the dominant lactic acid bacteria and potential pathogens as classified by the RDP Classifier (i.e. *Staphylococcus aureus*, *Bacillus cereus* group, *Klebsiella* sp.) were subjected to long-read WGS (Table 2, see next results section). In addition to the classical microbiology and sequencing analyses, we performed qPCR to quantify microbial DNA in the PBMA products. Given the methodical challenges and the heterogeneity of the sample types, we have detailed these qPCR results and the associated methodological considerations in the supplementary materials (Supplementary Notes 1).

**Table 2:** List of whole genome sequenced isolates

Species	Pathogen^1^	Fast ANI %^2^	Closest reference^3^	n^4^
Bacillales				
*Bacillus licheniformis*	x	99.55	GCF_000011645.1	4
*Bacillus paralicheniformis*	-	99.04	GCF_001042485.2	1
*Bacillus pumilus*	x	95.44	GCF_900186955.1	3
*Bacillus subtilis*	x	98.53	GCF_000009045.1	1
*Bacillus velezensis*	-	98.98	GCF_001461825.1	1
*Bacillus_A paranthracis*	-	97.47	GCF_001883995.1	3
Lactobacillales				
*Enterococcus_B faecium*	x	99.12	GCF_001544255.1	1
*Latilactobacillus sakei*	-	97.37	GCF_002370355.1	2
*Leuconostoc citreum*	-	97.84	GCF_004354555.1	1
*Leuconostoc mesenteroides*	x	98.88	GCF_000014445.1	3
Staphylococcales				
* Staphylococcus aureus*	x	98.93	GCF_001027105.1	1
Enterobacterales				
*Atlantibacter hermannii*	x	98.63	GCA_900635495.1	1
*Citrobacter braakii*	x	98.64	GCF_002075345.1	1
*Escherichia coli*	x	96.74	GCF_003697165.2	1
*Klebsiella grimontii*	x	99.26	GCF_900200035.1	1
*Klebsiella pasteurii*^5^	x	95.91	GCF_900200035.1	1
*Klebsiella oxytoca*	x	99.36	GCF_001598695.1	1
*Leclercia adecarboxylata*	x	98.41	GCA_901472455.1	1
*Lelliottia amnigena_A*	x	98.90	GCF_001652505.2	3
*Rahnella inusitata*	-	98.88	GCF_003263515.1	1

^1^documented established pathogen according to Bartlett et al.

^2^FastANI average nucleotide identity.

^3^ID of the closest placement reference.

^4^Number of whole genome sequenced isolates classified as this species.

^5^Species is not present in the GTDB, so the next reference genome is *Klebsiella grimontii.*

### Lactic acid bacteria and Gammaproteobacteria dominate PBMA products

**Fig. 2:**
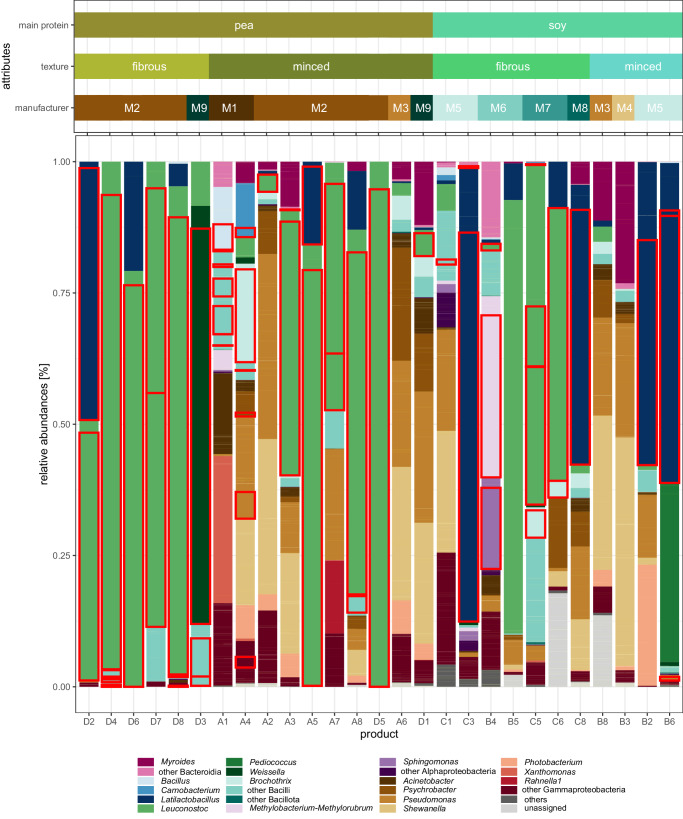
Taxonomy plot based on amplicon sequencing, showing the relative abundances on genus level. The underlying relative frequencies of the ASVs are recognizable as pale yellow lines within a genus. Genera with a maximum value of 10% across all samples were subsumed by color in the next higher taxonomic level. The samples are ordered by main protein source, texture and manufacturer. ASVs with matching isolates ( > 99% identity), were outlined in red.

In total, 27 samples (883,427 sequences; median frequency per sample: 26,083) sequenced by 16S rRNA gene amplicon HTS surpassed the quality criteria and proceeded to further downstream analysis. ASVs were assigned to 25 different phyla. Three of the samples showed > 3% relative abundance of at least one phylum (i.e. Bacillota 0.00-94.74%, Pseudomonadota 0.00–30.85%, Bacteroidota 0.00–10.84%). In total, 18 samples were dominated by Bacillota (10 samples with > 90% abundance) and the other 9 were dominated by Pseudomonadota. The most common genera were *Leuconostoc* (detected in 25 samples; ranging 0.03–100.00% relative abundance in the sample), *Latilactobacillus* (21 samples; 0.02-86.38%), *Pseudomonas* (20 samples; 0.36–35.25%), *Serratia* (19 samples; 0.03–8.92%), and *Acinetobacter* (17 samples; 0.09–15.40%). *Leuconostoc* was the most abundant genus in 13 samples, followed by *Latilactobacillus* (4 products), and *Shewanella* (4 products) (Fig. 2). Strikingly, some genera that showed a high relative abundance ( > 10%) in one or more sample were not able to be isolated by culture-dependent methods (i.e. *Shewanella*, *Xanthomonas*, *Photobacterium*, *Myroides*, *Pediococcus*).

**Fig. 3:**
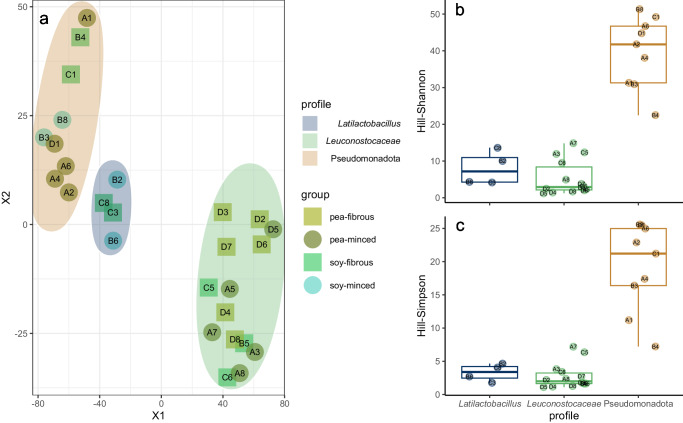
Differences in community profiles. **a** tSNE plot clustered samples to different profiles based on Bray-Curtis dissimilarity (Similar clusters were also found with other distance matrices - see Supplementary Fig. 2). **b** Hill-Shannon diversity and **c:** Hill-Simpson diversity comparing these profiles. Box plots show the median with hinges that correspond to the 25th and 75th percentiles. The whiskers extend from the hinge to the largest and smallest value no further than 1.5 multiplied by the inter-quartile range. Individual data points are overlaid.

The tSNE plot clearly showed three distinct clusters (Fig. 3a), which we described based on the most abundant taxa as *Leuconostocaceae*-, *Latilactobacillus*- and Pseudomonadota-profiles. The clustering was comprehensible, when comparing the similarity of the relative abundance patterns of the samples within each cluster. However, there was no clear separation based on the examined variables (main protein source, status, manufacturer). A Kruskal-Wallis test comparing the samples of the three clusters showed a significant difference for Hill-Simpson (*p* < 0.01) and Hill-Shannon (*p* < 0.01) diversity (Fig. 3b, c).

### Protein source and texture are not the main drivers for the microbial community pattern

**Fig. 4:**
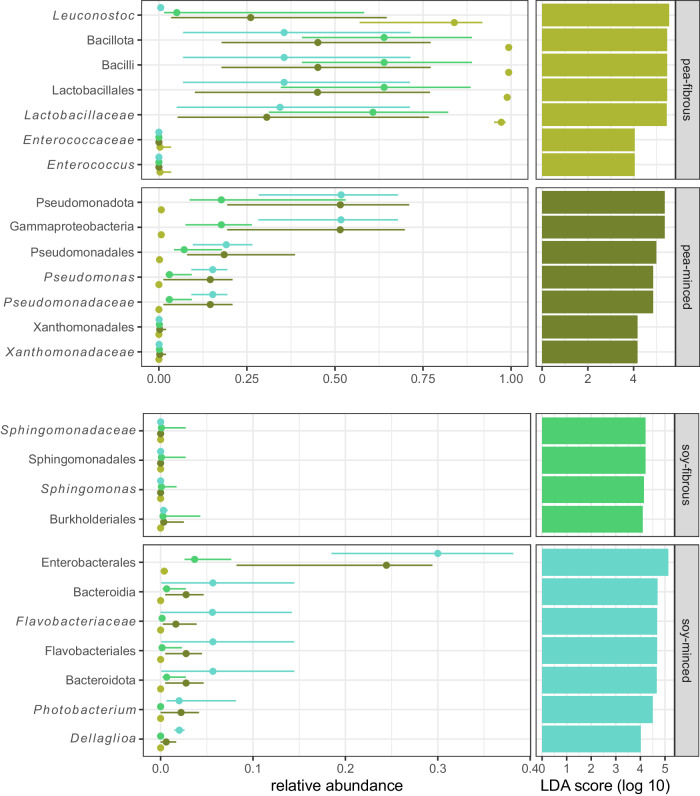
Linear discriminant analysis Effect Size (LEfSe) per group identified several predictive characteristic genomic features for product categories with a log_10_ LDA score > 4.0. Additionally illustrated are the medians of the relative abundances as a point, with lines representing the first and third quartiles. Additionally, LEfSe per community profiles are displayed in Supplementary Fig. 3.

The Kruskal-Wallis test comparing the Hill-Shannon index between the four groups (based on proteins source and texture) was significant (*p*-value = 0.02), while there was no significant difference comparing the group-wise Hill-Simpson index (*p* = 0.05). Post-hoc Dunn’s testing with Bonferroni alpha adjustment showed that when comparing group-wise Hill-Shannon index only the groups “pea-fibrous” and “pea-minced” differed significantly (*p* = 0.02 - Supplementary Fig. 1). The group dispersions were homogenous in all examined distance methods (i.e. Bray-Curtis, Jaccard, JSD). The PERMANOVA showed that texture and protein source significantly affected the microbial composition (Supplementary Table 1), but explained only between 15.94% and 23.44% of the total variance. The variance explanation by the PERMANOVA would increase, if the manufacturer as variable was added to the model, but as the sampling was very unbalanced, it was renounced. LEfSe identified several predictive characteristic genomic features for product categories with a log_10_ LDA score > 4.0. For “pea-fibrous” products the occurrence and abundance of *Leuconostoc* and *Enterococcus* were characteristic as well as the corresponding superordinate phylogeny levels. For “pea-minced” products it was mainly *Pseudomonas*. In “soy-fibrous” products two low abundant orders (i.e. Sphingomonadales and Burkholderiales) had a log_10_ LDA score > 4.0. The orders Enterobacterales and Flavobacteriales (Phylum Bacteroidota) were identified to explain microbial characteristics in this group (Fig. 4).

### Genomic features of isolates reveal potential microbiological risk

Most of the sequenced *Enterobacteriaceae* were described as established human pathogens according to Bartlett et al.^24^. Among these *Escherichia coli* was the only species, associated with foodborne diseases. The genome of the isolated *E. coli* strain revealed several virulence factors and antimicrobial resistance genes (AMRG) according to VFDB and CARD databases, respectively (Supplementary Table 2). This includes virulence factors like *fimH* (Type 1 fimbriae) and *fyuA* (ferric yersiniabactin uptake), which are associated with Adherent-Invasive *E. coli* (AIEC)^25^, or AMRG such as *mdfA* (associated with multidrug resistance) or *ampH* (beta-lactamase resistance). The three *Klebsiella* isolates belonged to the *K. oxytoca* species complex (KoSC) and were classified as *K. oxytoca*, *K. grimontii* and *K.pasteurii*. Different class A beta-lactamase genes were detected in the isolates, including *bla*_OXY-2_ in *K. oxytoca*, *bla*_OXY-6_ in *K. grimontii* and *bla*_OXY-4_ in *K. pasteurii*. Extended spectrum beta-lactamase (ESBL)- and carbapenemase-producing isolates of this species complex are associated with nosocomial infections^26^. The other *Enterobacteriaceae* isolates were classified as *Citrobacter braakii*, *Leclercia adecarboxylata* and *Lelliottia amnigena A*. Within the *Bacillus* genus, the 13 sequenced isolates were classified as *B. licheniformis* (*n* = 4), *B. pumilus* (*n* = 3), *B. paralicheniformis* (*n* = 1), *B. subtilis* (*n* = 1), *B. velezensis* (*n* = 1) and *B. paranthracis* (*n* = 3). Since *B. paranthracis* can cause foodborne illnesses^27^, their genomes were additionally analyzed with BTyper3 (Supplementary Table 2), which predicted the presence of the relevant non-hemolytic enterotoxin (Nhe) complex, which is associated with diarrhea, as well as other virulence factors. The *Staphyloccous aureus* genome harbored several virulence genes (Supplementary Table 2). The analysis of the lactic acid bacteria focused on spoilage potential (enzymatic repertoire for metabolite production) and preservative aspects. The sequenced *Leuconostoc* and *Latilactobacillus* isolates possessed genes to produce metabolites associated with meat spoilage, like butane-2,3-dione (diacetyl), 3-hydroxybutan-2-one (acetoin), 2-hydroxypropanoate (lactate), acetate and ethanol. One of the *Ln. mesenteroides* isolates harbored genes for a leucocin A/sakacin P family class II bacteriocin and the associated relevant transport proteins, while in *L. sakei* isolates only the transporter genes were detectable (antiSMASH hits and belonging blastp best hits in Supplementary Table 2). *Ln. mesenteroides* showed greater abilities for carbohydrate utilization in terms of glycoside hydrolases (26 GHs) in comparison to *L. sakei* (14 GHs). Further, *Ln. mesenteroides* harbored genes coding for enzymes in charge of synthesizing a broad range of amino acids (i.e. Threonine, Cysteine, Methionine, Valin, Isoleucin, Leucin, Arginin, Tryptophane) and menaquinone (Supplementary Table 2).

## Discussion

There is a lack of microbiological investigations for novel PBMAs, and although nutritional consequences, sustainability gains and sociotechnological pathways for transition to meat alternatives have been extensively reviewed (e.g. van der Weele et al. 2019^28^), currently microbiology, shelf-life optimization and risk assessments are currently lagging behind, considering the high variety of novel products in this category. Highly processed food, like the PBMAs examined in this study, present challenges to microbiologists. The broad range of ingredients (~120 in the 32 selected products), different processing steps, and a variety of production equipment may lead to many potential sources for spurious bacterial contamination. The product specific production processes were not available, but up to the present low (LMEC) and high moisture extrusion cooking (HMEC) are the most common commercially used technologies to produce meat textured plant proteins^29^. Both methods are based on an interaction of heat, shear force and pressure, but the conditions for the extrusion process depend on the original protein source and the desired final protein structure^30–32^. Looking at the survival abilities of microorganisms throughout this process, the crucial factors are temperature, pressure, and time. These variables are closely tied to the process configurations; hence, the following can merely serve as general guide. Specifically, within HMEC, the extruder barrels feature distinct temperature zones, with the high-temperature melting zone reaching approximately 150–170 °C^33^. However, the temperature at the extruder die is notably lower, being around 15–25 °C cooler than the barrel^34^. The barrel temperature for LMEC is general lower with 120–150 °C^35^. The pressures in these barrels is between 1 and 4 MPa in HMEC and up to 13 MPa in LMEC^35–37^. Yu et al.^38^ showed in different LMEC settings fastest particle residence times (first signs of the tracer) from 10 to 40 s and extrudate collection times (complete tracer passed) from 60 to 120 s, resulting in mean residence times of 35–87 s, depending on feed moisture, screw speed and die diameter. So far it is described that vegetative forms of bacteria are inactivated effectively by this process and only spores of bacteria like *Bacilli* and *Clostridia* would survive the extrusion process^39,40^. However, reactivation of spores during further processing is possible^41^. This could be one explanation why *Bacillus* was isolated from most of the samples (19/32), while the relative abundance of 16S rRNA gene DNA is relatively low, compared to other genera. Based on our results and the initial extrusion procedure, we can infer that the main protein would not be the primary source for the majority of the living bacterial cells we isolated from the products. The most likely causes of contamination are the addition of ingredients, specifically spices and herbs^42^, as well as the production environment, which includes the selection of operational equipment and handling procedures. In this context, future studies must focus on examining raw materials and production environments to confirm these findings and to adjust the production towards higher security and safety.

Based on the microbial distribution patterns from the 16S rRNA gene amplicon HTS data, we roughly can define three different bacterial community profiles. Lactic acid bacteria (LAB) dominated two of them (14/27 *Leuconostocaceae* dominated samples, 4/27 *Latilactobacillus* dominated samples). Interestingly, the majority of these samples, were dominated by one single ASV of these genera, which corresponded with the ones isolated. Based on WGS, the LAB representative isolates were classified as *Ln. mesenteroides*, *Ln. citreum* and *L. sakei*. *Ln. mesenteroides* and *Ln. citreum* are usually found in plants and plant-based foods^43^. *Ln. mesenteroides* is an important fermenter for products like kimchi, sauerkraut^44^, and soybean paste^45^. Additionally, they have shown antimicrobial activities against pathogenic bacteria, such as *Salmonella enterica* subsp. enterica serovar Typhimurium and *Listeria monocytogenes*^46,47^. On the other hand, *Ln. mesenteroides* has been also associated with meat spoilage, and the production of off-odors, off-flavours, slime and gas production^48–50^, particularly in combination with modified atmosphere packaging and cold storage^51–53^. Similar properties were attributed to *L. sakei*. It was described as fermenters particularly for sausages^54^, and vegetable products^55^, as producer of bacteriocins^56^, and as probiotics^57^. The detection of genes associated with the production of metabolites such as butane-2,3-dione (diacetyl), 3-hydroxybutan-2-one (acetoin), 2-hydroxypropanoate (lactate), acetate, and ethanol in the sequenced *Leuconostoc* and *Latilactobacillus* isolates might have important implications in the final organoleptic properties of this specific food products. Diacetyl and acetoin are volatile compounds that contribute to off-flavors and off-aromas in food products like meat when present in high concentrations. For example, these compounds impart buttery or metallic flavors and are often associated with undesirable sensory characteristics in spoiled meats^58^. The production of lactate, acetate, and ethanol indicates metabolic activities and also leads to changes in pH, acidity, and alcohol content^59^. These alterations affect the sensory attributes and quality, all potentially leading to spoilage. Metabolites produced by *Leuconostoc* and *Latilactobacillus* isolates, particularly diacetyl and acetoin, are also known to accelerate the deterioration of meat products, contributing to a shortened shelf life and reduced consumer acceptability, what could also be true for PBMAs, although further research is needed.

For kimchi and other plant products is described, that *Leuconostoc*, *Enterococcus* and *Lactococcus* act as initial colonizers, succeeded by *Lactobacillus*, *Pediococcus* and *Weissella*^43^. This can be explained by a better adaptability to environmental conditions (e.g. through a large number of accessory genes), but also by the already high population density on the ingredients, while in later stages decreasing pH values inhibits *Leuconostoc* species, which are sensitive to acids^43^. However, the examined products were very diverse in their shelf life, so it was difficult to conclude, whether *Latilactobacillus* dominated samples, were in a later stage of shelf life than the *Leuconostoc* dominated samples. In vegan meat alternatives, the drop in pH should not be comparable to kimchi (pH 4.0)^60^. It ranks in the range of 5.4–6.6^61,62^, and thus should not favor *L. sakei*. In general, in both species there are strains able to grow at 4 °C^51,55,63^, although *Ln. mesenteroides* strains examined by Comi and Iacumin grew faster at 4 °C than *L. sakei*^64^. *Leuconostoc* spp., among these *Ln. mesenteroides* and *Ln. citreum* are described to inhibit the growth of *L. sakei* strains^65^, but it was also demonstrated, that *L. sakei* is able to inhibit the growth of *Ln. mesenteroides*, when they were inoculated equally to cooked bacon^66^. However, *L.sakei* is highly adapted to protein rich meat and fish environments and so has reduced abilities for amino acid biosynthesis^67^. The *L. sakei* isolates from this study lack eight important amino acid biosynthesis pathways. However, these pathways were present in the *Ln. mesenteroides* isolates. Since most of these amino acids have lower concentrations in plant proteins compared to muscle proteins^68^, *Ln. mesenteroides* might have some growth advantages in PBMAs. Apart from this, the investigated *Ln. mesenteroides* isolates had more genes associated to the utilization of different carbohydrate sources (i.e. maltose, dextrins, isomaltose), compared to *L. sakei* (26 vs. 14 GHs). Additionally, the examined *Ln. leuconostoc* isolate had the ability to synthesise menaquinone (vitamin K2), which may mean better handling of oxidative and other environmental stressors and an increase in growth rate^69,70^.

Overall, we examined nine samples with high relative abundances of Pseudomonadota, mainly *Pseudomonas*, *Psychrobacter* and *Shewanella*. These three genera were described as common spoilers in meat and fish products^71^. Plant-based meat products are usually slightly higher in pH than their animal-derived counterparts^62^. Among the genera found in the 16S rRNA gene amplicon HTS, at least 13 of them are described as biofilm builders in food processing environments^72^, including *Pseudomonas*, *Psychrobacter* and *Shewanella*. The possibility of biofilm formation on the processing equipment is high, given that many of the machines used are difficult to clean. However, this notion is inconsistent with the fact that among the nine Pseudomonadota-dominated samples, seven exhibit very similar pattern, despite originating from six different producers. To our knowledge, there are no available studies on the microbial communities of raw soybean or pea proteins, but 16S rRNA gene patterns of peas or the phyllosphere of soy do not support the hypothesis that this kind of contamination is associated with the main protein source. Only sample B4, which is the sole sample dominated by Alphaproteobacteria, had high similarities to the relative abundances of the microbial community of the soy phyllosphere^73^. However, some of the producers just have a few products in this food segment, what could imply that they purchase already extruded proteins from a large distributor for use in their products. We assume that bacteria, which are metabolically active at some point during the process, contribute to the development of (off-)odors and (off-)tastes, and on the other hand, can provide information on whether there are contamination inputs from the ingredients or process areas that could be considered in the HACCP concept in the longer term (e.g. preventing biofilm formations).

Beside the dominant, spoilage associated genera, we also isolated different *Enterobacteriaceae* members including *Leclercia*, *Atlantibacter*, *Citrobacter*, *Escherichia* and *Klebsiella*. This goes in line with previous studies that showed the ability of certain bacteria to grow in vegan burgers^74^. The presence of *Enterobacteriaceae* in food samples is a hygiene indicator and usually associated with human handling and poor hygienic conditions. In this study, all *Enterobacteriaceae* isolates originated from products of the same manufacturer. 16S rRNA gene amplicon HTS revealed very low levels of *Enterobacteriaceae* in all products, suggesting that the heating processes during production effectively killed *Enterobacteriaceae* in these products, as previously reported^74^. All products were labelled with cooking instructions: most of them specifying cooking time in minutes, some only showing “heat through before consumption”. This final heating step by the consumer is considered as part of the HACCP concept of the manufacturer. While cooking time in minutes is a good guidance for the consumers, “heat through” is in our opinion too imprecise. First, there is a lack of experience with these kind of products, second in contrast to animal meat (products) there is no indicator like color change to determine a sufficiently cooked state. However, lack of experience with these products is not limited to preparation, but also to spoilage detection, handling of the raw product and shelf life or storage before and after preparation. In total, nine out of 32 samples had an expiry date, the others a best before date. Although the best-before date on these products is to be welcomed for reasons of sustainability, it is understandable that consumers are more inclined to discard the products once this date has passed, as they do not trust themselves to make an assessment. The odor of the examined products was not comparable to a corresponding meat product. Most of the products in this study were “ready-to-heat”, so there is no need for handling before the heating step. Additional preparation steps are most likely for vegan mince (mixing and forming steps). In this case, the same kind of kitchen hygiene is appropriate as is recommended for raw meat. Toth et al. concluded in their study that dishes with vegan meat substitutes spoiled faster than their meat counterparts when stored after preparation^62^. These findings, together with the generally higher refrigerator temperatures than recommended^75^, once again show the importance of increasing consumer awareness of food handling and storage. This is especially true, considering the potential pathogens isolated from the products. Taking into account the potential pathogens identified in the products, our research demonstrates that the detected *E. coli* strain carries virulence factors and antimicrobial resistance genes linked to AIEC, suggesting their potential to cause foodborne illnesses. For instance, the presence of *fimH*, responsible for encoding the FimH protein involved in bacterial adhesion, underscores the strain’s ability to adhere to host cells, facilitating infection establishment and biofilm formation^76^. Additionally, one strain harbored the multidrug resistance related gene *mdfA*, which might enhance the bacterium’s ability to withstand various antimicrobial agents^77^. Furthermore, the identification of *ampH* associated with resistance to beta-lactam antibiotics might limit treatment options in the case of potential colonization^78^. In summary, the presence of these virulence and antimicrobial resistance genes suggests that *E. coli* strains found in plant-based meat alternatives may possess heightened adhesive capabilities, increased survival mechanisms, resistance to multiple drugs, and resistance to specific classes of antibiotics, potentially posing challenges for infection treatment. Our detection of class A beta-lactamase genes, like *bla*_OXY-2_ in *Klebsiella oxytoca*, *bla*_OXY-6_ in *K. grimontii*, and *bla*_OXY-4_ in *K. pasteurii*, holds significant medical implications. These genes indicate the genetic capacity of the respective *Klebsiella* species to produce enzymes that can hydrolyze and inactivate beta-lactam antibiotics, such as penicillins and cephalosporins. This resistance mechanism poses a considerable challenge in treating infections caused by these bacteria, as beta-lactams are among the most used and prescribed antibiotics in clinical practice^79^. Consequently, healthcare providers are increasingly considering alternative antibiotic regimens or tailored therapies based on antimicrobial susceptibility testing results to ensure effective treatment^80^. The *Staphylococcus aureus* genome harbored several AMRG, such as *norA* (associated with resistance to quinolones) and *tet(38)* associated with resistance to tetracyclines), that contribute to their ability to survive in the presence of antimicrobial agents^81,82^. Other AMRG, such as *mepA* and *lmrS*, were also found in the *S. aureus* genomes, and have been previously reported to be part of the host innate immune defenses^83,84^. The targeted WGS analysis performed here gave a first direct hint to the potential of some PBMA products for causing foodborne diseases.

Regarding the potential drivers of microbial composition in PBMAs we showed that main protein source and texture were significant variables in the PERMANOVA, but they only explained about 18% of the model’s variance. Adding the manufacturer as additional variable to the PERMANOVA increased the explained variance to 53%. Even if our sample selection was too unbalanced in terms of manufacturers to be able to make valid statistic statements we assume that the production plant has an non-negligible effect on the product’s microbial community. The common perception of the public that plant-based is inherently healthy, must be seen critically. As with any other food product, the macro-nutrient profile and the level of processing are important factors to be considered when determining how healthy a product is. It’s important for producers to label carefully and with accessible wording (as explanations are read by the general public), so that consumers can understand what they are purchasing and consuming. However, balancing plant-based meat alternatives with whole, minimally processed foods like vegetables, fruits, grains, nuts, and legumes optimizes nutritional value, and this message should be communicated as well.

A combination of culture techniques and various sequencing methods served to provide insights into the microbiology of the product group of PBMAs that were hardly available until now. The results show a clear dominance of lactic acid bacteria (i.e. *Leuconostoc mesenteroides* and *Latilactobacillus sakei*) in most of the sampled products, whose genetic profile suggests that they are the main culprits for the spoilage of these products. In the long term, a more detailed study of these species on these products could lead to an improvement in shelf life. In addition, several species were isolated that may be associated with foodborne illnesses (e.g. non-hemolytic enterotoxin genes possessing *Bacillus paranthracis*, *Staphylococcus aureus*, *Escherichia coli*). The presence of these species in this relatively small set of samples shows that this product group may well serve as a medium for foodborne pathogens if kitchen hygiene is lacking or recommended heating steps are missing, imprecisely formulated, or overlooked. Further, targeted investigations of larger samples groups would be valuable.

## Methods

### Sample acquisition

We purchased 32 different PBMAs, between July 12 and July 14, 2021, from four leading supermarket chains in Vienna, Austria. The focus was on pea- and soybean- protein based products with either a minced or a fibrous texture, since they have been the most common representatives of PBMA in Austria. Additional criteria for the selection included that the products were entirely plant-based (vegan) and did not contain fermented products, like tofu. Beside these characteristics, the samples were different in their composition, packing, shelf life, etc. (Table 1). All samples were transported refrigerated and stored at 4 °C until their immediate processing.

### Sample preparation

In total, 10 g of each sample were homogenized 120 s with 90 ml sterile 1 × phosphate buffered saline (PBS, Gibco, Bleiswijk, The Netherlands) in sterile Stomacher^®^400 classic strainer bags (Seward Ltd, Worthing, United Kingdom) using a BagMixer^®^400 CC (Interscience, Puycapel, France). To remove coarse food particles, the homogenates were centrifuged at 300 × rcf (relative centrifugal force) for 2 min at room temperature (RT) using an Eppendorf Centrifuge 5810R and an A-4-62 rotor (Eppendorf Corporate, Hamburg, Germany). The remaining supernatants were transferred to new tubes and centrifuged at 3000 × rcf (30 min at RT). The obtained cell pellets were diluted 1:10 (v/v) with sterile 1 × PBS and were used freshly for bacterial and fungal isolation or were frozen at −20 °C for later cultivation approaches and at −80 °C for DNA extraction.

### Bacterial and fungal cultivation

We carried out two sets of cultivation experiments, i) broad range and ii) targeted cultivation. To get a broad range of isolates from the expected microbial communities, the first set was done with several non-selective and selective media (Columbia agar (BioMérieux, Marcy l’Etoile, France), violet red bile glucose agar (VRBG, Biokar diagnostics, Allonne, France), brain heart infusion agar (BHI broth (Biokar diagnostics) + 1.5% agar (bacteriological agar type E, Biokar diagnostics)), plate count agar (PCA, Biokar diagnostics), Rose Bengal chloramphenicol agar (Biokar diagnostics), and Baird Parker agar (GranuCult™, Merck, Darmstadt, Germany)). The plates were inoculated with 100 *μ*l of a 10^−^^2^ dilution of the sample and incubated at 37 °C both aerobically and semi-anaerobically (using GENbox anaer, BioMérieux, in BD BBL™ GasPak™ systems, Becton Dickinson, New Jersey, United States). In order to recover high amounts of different species, the plates were incubated for 16–68 h, depending on colonies’ sizes and growth densities. The primary goal of our cultivation process was to isolate a diverse range of microbial species, rather than to quantify or describe the microbial composition in detail. Consequently, when we encountered samples exhibiting overly dense growth after 16 h of incubation, which precluded the selection of individual colonies, we proceeded to further dilute the samples 10^−^^3^ or 10^−^^4^ in sterile 1 × PBS and plate these anew. Given our focus on maximizing species diversity, any potential changes in microbial community composition or growth patterns during this period were deemed secondary concerns. For the targeted cultivation, we leveraged the insights from 16S rRNA gene amplicon sequencing to tailor specific media and growth conditions aimed at isolating representatives of genera not captured under initial culturing conditions. This strategy was facilitated by utilizing cell pellets that had been preserved at −20 °C, enabling us to revisit and effectively target these genera several months after the initial sequencing efforts. Depending on the sample specific microbial composition, we used Luria Bertani agar (1% tryptone (Oxoid, Basingstoke, UK), 0.5% yeast extract (micro-granulated, Roth, Karlsruhe, Germany), 1% NaCl (Sigma-Aldrich, St. Louis, USA), 2% agar, pH 7.0), nutrient agar (0.5% casein peptone, tryptic digest (Roth), 0.3% beef extract powder (Fluka analytical, Seelze, Germany), 1.5% agar, pH 7.0), trypto-casein soy agar (TSA,Biokar diagnostics), marine agar (marine broth (Roth) + 1.5% agar), corynebacterium agar (1% casein peptone, tryptic digest, 0.5% yeast extract, 0.5% D(+)-glucose (Roth), 0.5% NaCl, 1.5% agar, pH 7.3), De-Man-Rogosa-Sharpe agar (MRS, Oxoid) and pseudomonas agar F (1% tryptone, 1% casein peptone, 0.15% K_2_HPO_4_, 0.15% (Roth), MgSO_4_ (Merck), 1% glycerol (Roth), 1.5% agar) and cultivated the samples (dilution 10^2^–10^5^) aerobically at 25 °C for 48 h.

### Isolate identification and whole genome sequencing

In both cultivation experiments, we selected morphologically unique, single colonies for re-cultivation followed by 16S rRNA gene Sanger sequencing of the pure cultures for identification. DNA was extracted, using a protocol modified after Walsh et al.^85^, by lysing pure cultures with 100 *μ*l 0.01 M TRIS/HCl (Trizma^®^base, Sigma-Aldrich, St. Louis, United States) and 400 *μ*l 2.5% Chelex^®^100 Resin solution (BioRad, Hercules, United States) at 95 °C for 10 min, followed by centrifugation with 15,000 × rcf for 3 s. The supernatants were subsequently used for 16S rRNA gene PCR, using a final concentration of 200 nM of each of the universal primers from LGC Genomics GmbH (Berlin, Germany; 27F – 5’-GAG TTT GAT C**M**T GGC TCA G-3’ and 1492R – 5’-GG**Y** TAC CTT GTT ACG ACT T-3’), 0.025 U/*μ*l Platinum™ Taq DNA-Polymerase (Invitrogen™, Vilnius, Lithuania), 1 × TaqMan PCR buffer, 2 mM MgCl_2_, and 250 *μ*M dNTP Mix (Thermo Scientific™, Vilnius, Lithuania). For the PCR a protocol of 95 °C for 5 min (Taq activation) followed by 35 cycles of 40 s at 95 °C (denaturation) 40 s at 52 °C (annealing) and 1 min at 72 °C (elongation) was used. Negative extraction and PCR controls were included in the experiments. In-house *Listeria monocytogenes* DNA served as a positive control. All PCR products were checked with a QIAxcel DNA High Resolution Kit (Qiagen, Hilden, Germany) in the QIAxcel Advanced system (Qiagen). Samples without detectable amounts of PCR products were used for an ITS2 region PCR (200 nM of each of the primers ITS3 – 5’-GCA TCG ATG AAG AAC GCA GC-3’ and ITS4 – 5’-TCC TCC GCT TAT TGA TAT GC-3’ (White et al. 1990), 0.025 U/*μ*l Platinum™ Taq DNA-Polymerase (Invitrogen™), 1 × TaqMan PCR buffer, 2 mM MgCl_2_, and 250 *μ*M dNTP Mix (Thermo Scientific™), with a protocol of 95 °C for 5 min (Taq activation) followed by 30 cycles of 40 s at 94 °C, 40 s at 56 °C and 1 min at 72 °C). For cultures negative in 16S rRNA and ITS2 PCR, we repeated the extraction with the NucleoSpin™ Tissue kit (Machery-Nagel, Düren, Germany), using the manual in combination with the recommendations for hard-to-lyse bacteria. LGC Genomics GmbH purified and Sanger sequenced the PCR products in one direction (using 27F primer for 16S rRNA and ITS4 for the ITS2 region). Potential pathogens (*n* = 15) and unclassified *Enterobacteriaceae* (*n* = 6) were further subjected to whole genome sequencing (WGS) by using FLO-MIN106 flow cells on a MinION Mk1C (Oxford Nanopore Technologies, Oxford, UK). The library preparation for this approach was done according to the protocol of Oxford Nanopore Technologies ("Ligation sequencing gDNA - native barcoding (SQK-LSK109 with EXP-NBD196)”^86^) using the NEBNext^®^ FFPE DNA repair kit (New England BioLabs^®^ Inc., Ipswich, United States) for DNA repair and end-preparation, NEB Blunt/TA Ligase Master Mix (New England BioLabs^®^ Inc.) and Native Barcoding Kit 96 (EXP-NBD196, Oxford Nanopore Technologies), for native barcode ligation, Adapter Mix II (Oxford Nanopore Technologies) and NEBNext^®^ Quick Ligation Module (New England BioLabs^®^ Inc.), for adapter ligation, Agencourt AMPure XP beads (Beckman Coulter™), for clean-up steps and SQK-LSK109 sequencing kit (Oxford Nanopore Technologies).

### 16S rRNA gene amplicon sequencing

For direct DNA extraction from PBMA samples the DNeasy^®^ PowerFood^®^ Microbial Kit (Qiagen) was used. For that purpose, the cell pellets stored in PBS (−80 °C) were thawed on ice, centrifuged (3000 × rcf, 30 min) and resuspended in 450 *μ*l MBL buffer. Deviating from the DNeasy^®^ PowerFood^®^ Microbial Kit Handbook^87^, the lysis step was proceeded in Lysing Matrix A, 2 ml tubes (MP Biomedicals Germany GmbH, Eschwege, Germany). 16S rRNA gene amplicon library generation and sequencing was performed at the Vienna Biocenter Core Facilities NGS Unit (Vienna, Austria, www.vbcf.ac.at). Sequencing libraries of the 16S rRNA gene (V3/4 region) were prepared based on Illumina 16S rRNA Gene Amplicon Sequencing Library Preparation recommendations. Primers 341F (5’-CCT ACG GG**N** GGC **W**GC AG-3’) and 805R (5’-GAC TAC **HV**G GGT ATC TAA TCC-3’) (Klindworth et al. 2013) were used together with Illumina adapter sequences (5’-CGT CGG CAG CGT CAG ATG TGT ATA AGA GAC AG-3’ and 5’-GTC TCG TGG GCT CGG AGA TGT GTA TAA GAG ACA G-3’, respectively) for amplification. Libraries were constructed by ligating sequencing adapters and indices onto purified PCR products using the Nextera XT Sample Preparation Kit (Illumina). Equimolar amounts of each of the purified amplicons were pooled and sequenced on an Illumina MiSeq Sequencer with a 300 bp paired-end read protocol, yielding a median of 96,965 sequences per sample.

### Sequence processing and statistics

The raw sequencing data obtained from the MiSeq platform was analyzed by using the QIIME 2 v2021.4.0^88^ pipeline. The first step involved quality control, denoising, paired-end merging, chimera removal and inference of amplicon sequence variants (ASV) by using DADA2^89^. The taxonomic classification was done with the Scikit-learn algorithm using a pre-trained full-length-uniform-classifier based on the SILVA 138.1 database, by using the q2-feature-classifier plugin^90–94^. After the removal of sequences classified as “mitochondria” or “chloroplast”, the resulting ASV table was imported into R v4.1.0 environment^95^ for further downstream analysis. To estimate the alpha diversities of the samples Hill-Simpson and Hill-Shannon diversity were calculated using the “iNEXT” v3.0.0 package^96^, based on 99.5% coverage rarefied samples^97,98^. Group comparisons were done with Kruskal-Wallis tests followed by Bonferroni-alpha-corrected Dunn’s tests for pairwise comparisons. For beta diversity analyses the samples were rarefied with 100 iterations depending on a coverage of 99.5% using the package “phyloseq” v1.38.0^99^ and “metagMisc” v0.0.4^100^. Based on that we generated distance matrices with Bray-Curtis dissimilarities, Jaccard indices and Jensen-Shannon divergence (JSD) and use them for graphical (t-distributed stochastic neighbor embedding - tSNE) and statistical (PERMANOVA, LEfSe) analysis. For tSNE we used the “Rtsne” v0.16 package^101^ with a maximum of 999 iterations, a perplexity of 5 and two initial dimensions, as recommended by Oskolkov^102^. The high variability of the products and little knowledge on the underlying production conditions made it difficult to identify variables with adequate explanatory power for PERMANOVA, besides the main protein source and the texture of the PMBA products. The producing facility as additional variable would be meaningful, but the product assortment is dominated by one company, which made the model design very unbalanced. PERMANOVA was done with the “vegan” v2.6.4 package^103^ using “betadisp” to check for homogeneous dispersion and “adonis” functions for PERMANOVA with 999 iterations using main protein source and texture as explanation variables. Linear discriminant analysis Effect Size (LEfSe) was done with the relative abundance data of the coverage rarefied data in combination with “phyloseqCompanion” v1.1 package^104^ for data transformation, followed by the use of the LEfSe Bioconda tool by Segata et al.^105^, using the protein source and texture as class, a normalization of 10^6^ and a log_10_ LDA score threshold of 4.0. In parallel, a group comparison for the same features, as examined with LEfSe, was done with Kruskal-Wallis tests followed by a Benjamini-Hochberg alpha correction.

The Sanger sequences from the isolates were trimmed using the “SangerRead” function within the “sangeranalyseR” v1.4.0 package^106^ with a Phred score mean quality cutoff of 40 and a sliding window size of 15 bp. Trimmed sequences, with > 100 bp length, were classified with the “assignTaxonomy” function of the “dada2” v1.22.0 package^89^ in R (with k-mer size 8 and 50 bootstrap replicates), based on an RDP (Ribosomal Database Project) Naïve Bayesian Classifier algorithm^107^. Further, the isolate sequences were assigned to a database generated from the MiSeq data set to connect the culture-based and culture-independent approaches. For Fig. 1, the isolate sequences of each genus were clustered within each sample, group and producer using the “IdClusters” function from the “DECIPHER” v2.22.0 package^108^ with a cutoff of 0.06. More clusters within each genus were interpreted as a higher species or strain diversity within each genus.

The WGS data produced with the MinION platform was trimmed and filtered with Filtlong v0.2.1^109^ and assembled with Flye v2.9^110,111^. Several polishing steps (four repetitions of Racon v1.5.0^112^ and a final step with Medaka v1.6.0^113^) followed, and the resulting genomes were analyzed by using the TORMES v1.3.0 ^114^ pipeline with the default parameters as follows: Assembly statistics was performed by using QUAST^115^. The taxonomy of the genomes was inferred by using Kraken2^116^ and by extraction of the 16S rRNA genes by using Barrnap v0.9^117^ and further classified by using the RDP Classifier^107^. Multi-locus sequence typing (MLST) was performed by using mlst v2.19.0^118^. CDS prediction and protein annotation was performed by using Prodigal v2.6.3 ^119^ and Prokka v1.14.6^120^. Antimicrobial resistance and virulence genes screening was performed by using Abricate v1.0.1^121^ against the comprehensive antibiotic resistance database (CARD)^122^ and the virulence factor database (VFBD)^123^, respectively. Further classification of the assembled genomes was performed by using the GTDB-Tk v2.2.0+^124,125^ and rMLST^126^. BTyper3^127^ was used for isolates classified with GTDB-Tk as *Bacillus paranthracis*. Lactic acid bacteria with good quality genomes were additionally examined with BlastKOALA v2.3^128^, dbCAN3^129^, combined with the databases KEGG (Kyoto Encyclopedia of Genes and Genomes https://www.kegg.jp/) or CAZymes (Carbohydrate Active Enzymes database http://www.cazy.org/)^130^, respectively, and antiSMASH v6.1.1^131^. The genes within clusters predicted with antiSMASH where then confirmed using protein-protein Basic Local Alignment Search Tool (BLAST®)^132^. All tools were used with default settings if not mentioned otherwise.

### Reporting summary

Further information on research design is available in the Nature Research Reporting Summary linked to this article.

### Supplementary information

Supplementary Information

Supplementary Table 2

Reporting Summary

## Data Availability

All sequencing data supporting the findings of this study are openly available in the following repositories: 16S rRNA gene amplicon sequencing data and MinION whole genome sequencing data of microbial isolates from food samples have been deposited in the European Nucleotide Archive (ENA) under the study accession number PRJEB73555. The Sanger sequencing data, presented in FASTA format, are available on GitHub at https://github.com/ffroch/veggiemeat.

## Code availability

All used codes are available on GitHub at https://github.com/ffroch/veggiemeat. This repository includes detailed documentation on the data processing and analysis methods used in this study.
