# Supplementary material for: Microbial community structure of plant-based meat alternatives
Source: NPJ Sci Food. 2024 May 13;8:27. doi: 10.1038/s41538-024-00269-8 (PMC11091099; doi:10.1038/s41538-024-00269-8)
Supplement: Supplementary file 1 — Supplementary Information [file 41538_2024_269_MOESM1_ESM.pdf]

Supplementary Figure 1

Groupwise comparison of the alpha-diversity using Hill-Shannon and Hill-Simpson indices. Hill-Shannon index differed significantly between fibrous and minced pea products ($p = 0.016$). Box plots show the median with hinges that correspond to the 25th and 75th percentiles. The whiskers extend from the hinge to the largest and smallest value no further than 1.5 multiplied by the inter-quartile range. Individual data points are overlaid.

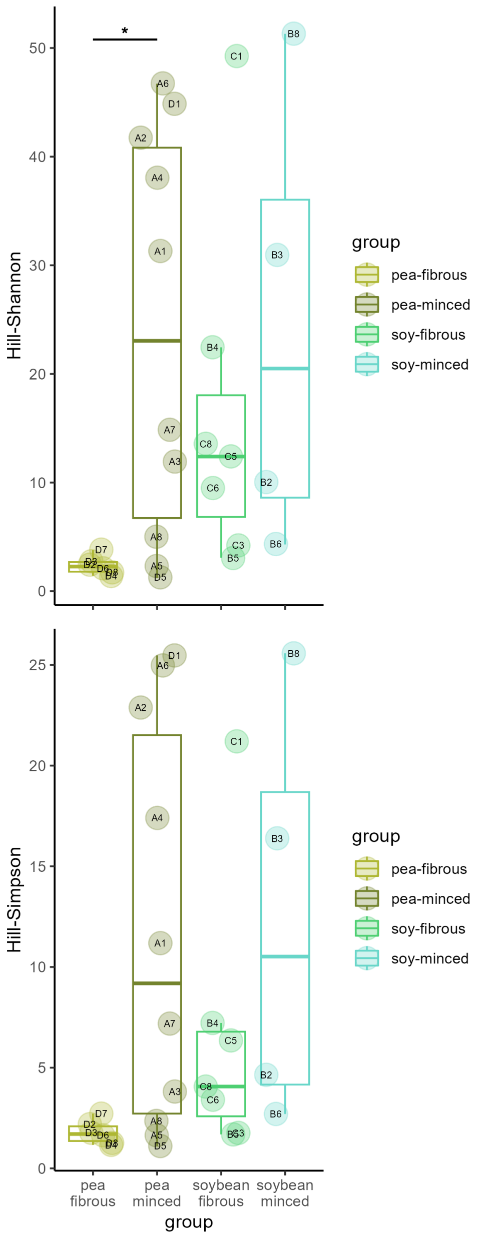

Supplementary Figure 2

tSNE plots based on Bray-Curtis dissimilarity, Jaccard distance and Jensen-Shannon divergence. In all three methods, the same clusters form.

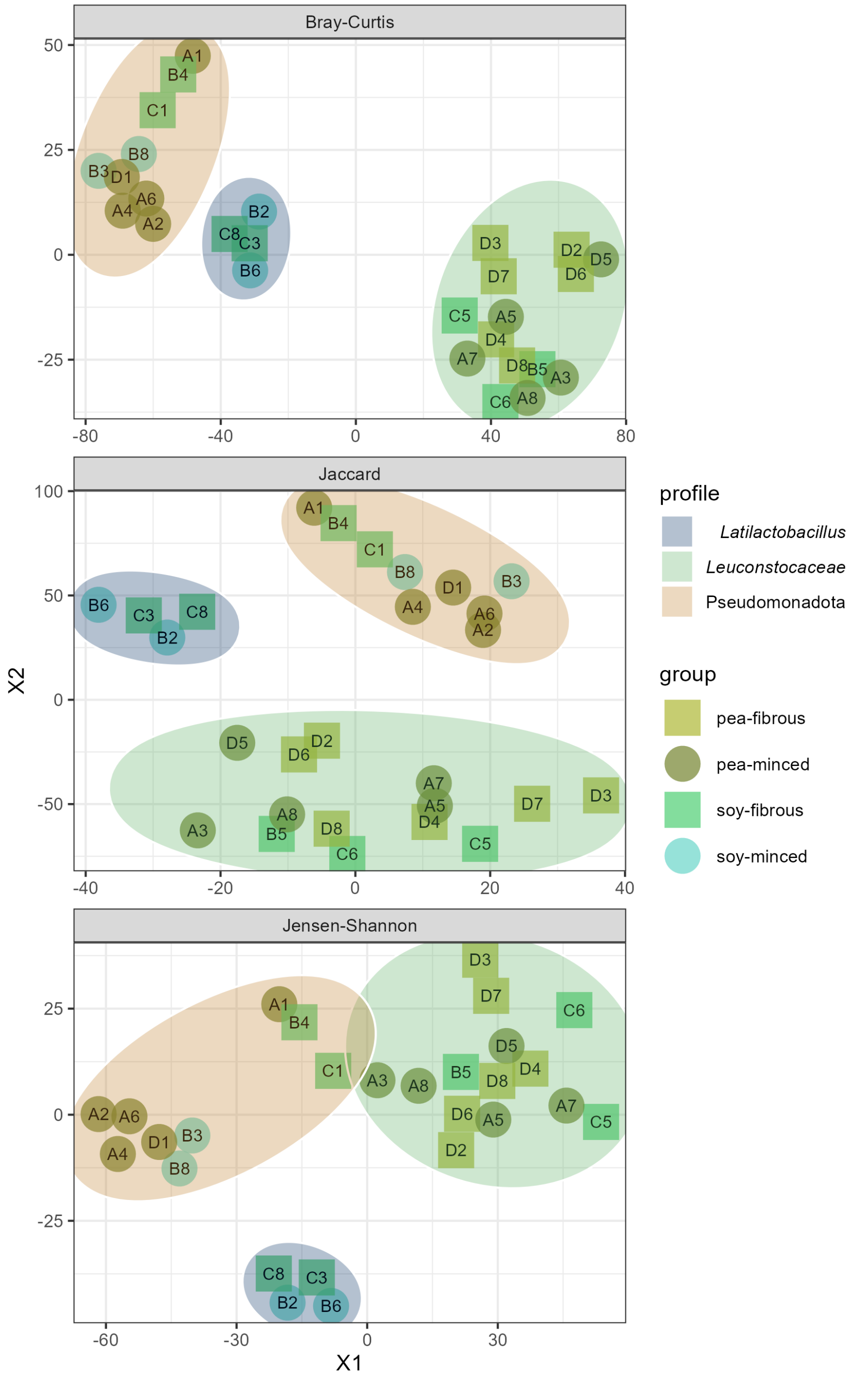

Supplementary Figure 3

LEfSe per profile. Additionally illustrated are the medians of the relative abundances as a point, with lines representing the first and third quartiles.

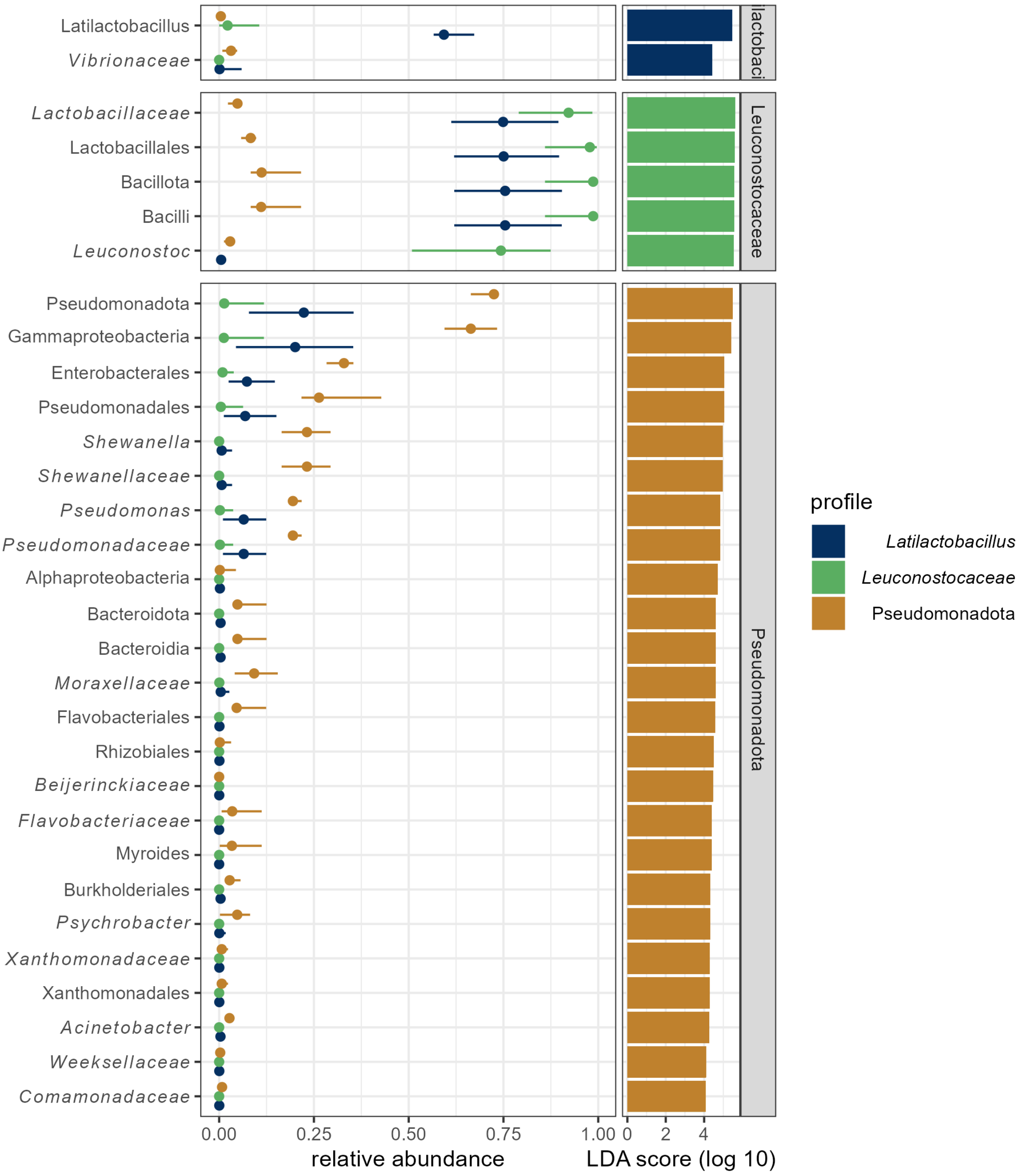

Supplementary Table 1

PERMANOVA results based on different distance matrices.

	Df	Sum of Sqs	R2	F	Pr. F
**Bray-Curtis**					
Permutation test for adonis under reduced model					
Terms added sequentially (first to last)					
Permutation: free					
Number of permutations: 999					
*adonis2(formula = dmlistshort[[i]] ~ protein.source * texture,*					
data = data.frame(sample_data(relab_po)), permutations = 999)					
protein source	1	0.7258	0.0743	2.090	0.030
texture	1	0.8391	0.0859	2.416	0.017
protein source:texture	1	0.2185	0.0224	0.629	0.825
residual	23	7.9874	0.8175		
total	26	9.7708	1.0000		
Permutation test for adonis under reduced model					
Terms added sequentially (first to last)					
Permutation: free					
Number of permutations: 999					
*adonis2(formula = dmlistshort[[i]] ~ texture * protein.source,*					
data = data.frame(sample_data(relab_po)), permutations = 999)					
protein source	1	0.6784	0.0694	1.953	0.041
texture	1	0.8864	0.0907	2.553	0.017
protein source:texture	1	0.2185	0.0224	0.629	0.834
residual	23	7.9874	0.8175		
total	26	9.7708	1.0000		
**Jaccard**					
Permutation test for adonis under reduced model					
Terms added sequentially (first to last)					
Permutation: free					
Number of permutations: 999					
*adonis2(formula = dmlistshort[[i]] ~ protein.source * texture,*					
data = data.frame(sample_data(relab_po)), permutations = 999)					
protein source	1	0.6774	0.0626	1.712	0.041
texture	1	0.7484	0.0691	1.891	0.022
protein source:texture	1	0.3001	0.0277	0.758	0.789
residual	23	9.1035	0.8406		
total	26	10.8295	1.0000		
Permutation test for adonis under reduced model					
Terms added sequentially (first to last)					
Permutation: free					
Number of permutations: 999					
*adonis2(formula = dmlistshort[[i]] ~ texture * protein.source,*					
data = data.frame(sample_data(relab_po)), permutations = 999)					
protein source	1	0.6367	0.0588	1.609	0.065
texture	1	0.7891	0.0729	1.994	0.014

Table 1: PERMANOVA results based on different distance matrices. *(continued)*

	Df	Sum of Sqs	R2	F	Pr. F
protein source:texture	1	0.3001	0.0277	0.758	0.770
residual	23	9.1035	0.8406		
total	26	10.8295	1.0000		
**Jensen-Shannon**					
Permutation test for adonis under reduced model					
Terms added sequentially (first to last)					
Permutation: free					
Number of permutations: 999					
*adonis2(formula = dmlistshort[[i]] ~ protein.source * texture,*					
data = data.frame(sample_data(relab_po)), permutations = 999)					
protein source	1	0.3254	0.0874	2.626	0.034
texture	1	0.4556	0.1224	3.676	0.008
protein source:texture	1	0.0918	0.0246	0.740	0.607
residual	23	2.8504	0.7656		
total	26	3.7231	1.0000		
Permutation test for adonis under reduced model					
Terms added sequentially (first to last)					
Permutation: free					
Number of permutations: 999					
*adonis2(formula = dmlistshort[[i]] ~ texture * protein.source,*					
data = data.frame(sample_data(relab_po)), permutations = 999)					
protein source	1	0.3698	0.0993	2.984	0.019
texture	1	0.4111	0.1104	3.318	0.007
protein source:texture	1	0.0918	0.0246	0.740	0.609
residual	23	2.8504	0.7656		
total	26	3.7231	1.0000		

Supplementary Table 2

Please check the attached .xlsx file

Supplementary Notes 1

Methods: Standard curves for quantification of bacterial 16S rRNA gene copy numbers were constructed by using the same primer set to amplify serial dilutions of purified PCR products from all sample types. Briefly, aliquots of extracted gDNAs (see section "16S rRNA gene amplicon sequencing") from all 32 samples were pooled and subjected to conventional PCR amplification. The PCR reaction included a final concentration of 180nM for each of the universal primers (5'-CCT ACG GG**D** GGC **W**GC A-3', 5'-GGA CTA C**HV** GGG TMT CTA ATC-3'; Eurofins Genomics, Ebersberg, Germany), 0.075 U/μl Platinum^{TM}Taq DNA-Polymerase (Invitrogen^{TM}, Vilnius, Lithuania), 1 × TaqMan PCR buffer, 3.5 mM $MgCl_2$, and 200 μM dNTP Mix (Thermo Scientific^{TM}, Vilnius, Lithuania). The PCR protocol consisted of an initial Taq activation step at

95°C for 10 min, followed by 40 cycles of denaturation at 95°C for 15 s, and an annealing/elongation step at 60°C for 60 s. The PCR was performed using a Bio-Rad T100™Thermal Cycler (Bio-Rad Laboratories (Singapore) Pte. Ltd., Republic of Singapore). The resulting PCR product was purified using an innuPREP PCRpure Kit (IST Innuscreen GmbH, Berlin, Germany). Subsequently, the purified PCR product was quantified using Qubit™ 2.0 Fluorometer (Life Technologies Corporation, Carlsbad, California United States) in combination with the Invitrogen Qubit™ dsDNA BR Assay Kit (by Thermo Fisher Scientific, produced by Life Technologies Corporation, Eugene, Oregon United States). Afterwards, the purified PCR product was subjected to a 10-fold serial dilution ranging from 10^{0}-10^{-6} to create an in-run standard curve.

To assess the absolute abundance differences in the samples, the level of total bacteria was quantified using the TaqMan® BactQuant assay targeting the V3–V4 regions of the gene (Liu et al., 2012). The assay is based on a conserved region of the 16S rRNA gene (466 bp) with forward 5'-CCT ACG GGD GGC **W**GC A-3' and reverse primers 5'-GGA CTA C**HV** GGG TMT CTA ATC -3' as well as the probe 6-FAM-5'-CAGCAGCCGCGGTA-3'-MGBNFQ (Eurofins Genomics). Each DNA food sample was run in duplicate in a final volume of 20μl reaction mixture, using 0.2 mL optical tubes sealed with MicroAmp optical 8-cap strips (Applied Biosystems). Single amplification reaction consisted of DEPC-treated water, 1 $\times$ TaqMan PCR buffer, 3.5 mM $MgCl_2$, 180 nM (final concentration) of each universal primer, a probe with the concentration of 225 nM, 0.075 U/μl Platinum™Taq DNA-Polymerase (Invitrogen™), 200 μM dNTP Mix (Thermo Scientific™) and template DNA. The quantification of DNA was performed in Mx3000P™ qPCR Multiplex Quantitative PCR System (Agilent Stratagene®, Santa Clara, California United States) (software v.4.10) after initial denaturation at 94°C for 10 min, followed by 40 cycles of 94°C for 15 s, 60°C for one min. Negative extraction and PCR negative controls were included in each qPCR run. Additionally, to detect potential inhibition of the DNA, samples were diluted (10-fold, 100-fold, 1000-fold) to determine the appropriate dilution range for the extracted samples.

The number of bacterial 16S rRNA genomic equivalents per g food (BCE/g) present in a sample was calculated from Ct by using a standard curve. The final copy numbers of total bacteria were calculated using a mean of the copy number per g food, including the calculation of the DNA volume subjected to qPCR, the volume of extracted DNA, and the weight of the sample subjected to the DNA extraction. An additional correction based on the 16S rRNA amplicon sequencing data was done with R v4.1.0 (R core Team, 2021) and the Biostrings package v2.62.0 (Pagès et al., 2021). For graphical display and description, R was used in combination with the ggplot2 package v3.3.6 (Wickham, 2016).

Results and Discussion: In the raw data of the 16S rRNA amplicon sequencing we observed that some

samples contained a significant amount of chloroplast and mitochondrial DNA. Since the primers used for qPCR are very similar to those used for 16S rRNA sequencing (fwd 16S rRNA sequencing: 5'-CCT ACG GG**N** GGC **W**GC AG-3') fwd qPCR: 5'-CCT ACG GG**D** GGC **W**GC A-3', rev 16S rRNA sequencing: 5'- GAC TAC **HV**G GGT ATC TAA TCC-3‘, rev qPCR: 5'-GGA CTA C**HV** GGG T**M**T CTA ATC-3'), we suspected that non-bacterial DNA could also be quantified in qPCR. Therefore, we analyzed the ASVs classified as chloroplasts or mitochondria to determine if they could also bind to the qPCR probe. This enabled us to determine the proportion of ASVs to which the probe bound, allowing for the necessary correction of the qPCR results.

In most cases, this correction had only a minimal effect (Addon 1) on the results. Nevertheless, we recognized that quantifying mitochondria and chloroplasts could be potentially problematic, particularly in the food sector, and would warrant a product-specific evaluation. For quantification, we did not rely on bacterial cultivations, but instead made semi-quantitative statements based on cultivation on Columbia Blood Agar plates. As described in section 1 of the main manuscript, samples were plated at a dilution of $10\hat{2}$. Plates with no or only a few colonies (up to 10) were classified as negative or very low. Plates that were moderately overgrown, allowing most colonies to be individually picked (i.e., up to 300 colonies) were labelled as medium. Samples that were heavily overgrown after 16 h and required re-plating were classified as high. This rough estimation aligned well with the quantification using qPCR (Addon 1).

The number of genomic equivalents detected in the samples ranged from "under detection limit" to log 7.51 BCE/g. Notably, two samples (B3 and C1) showed significant amounts of bacterial genomic DNA in the qPCR but showed limited growth in cultivation. This discrepancy highlights the limitations of both qPCR and quantification by cultivation. In our experience, qPCR typically detects 10-100 times more genomic equivalents than colony-forming units in cultivation. This discrepancy can be attributed to qPCR's ability to quantify dead cells and the fact that not all bacteria can be successfully cultivated, especially using general standard quantification methods. However, in the food sector, particularly for highly processed products, two other factors play a critical role. Firstly, certain steps in the production process can lead to the mass death of the bacterial community, such as heating steps. As a result, a substantial amount of bacterial DNA may still be present, even though few viable cells are remaining. Secondly, various processes or changes in conditions such as cooling or packaging in a modified atmosphere can result in the presence of viable but not culturable (VBNC) bacteria. These bacteria are alive and often metabolically active but cannot be cultivated under standard conditions. Consequently, their presence can lead to inaccurately low results in quantification through cultivation (Ayrapetyan and Oliver, 2016). Individual factors or a combination of factors could have

contributed to these discrepancies. As a result, of these limitations, we have refrained from using statistical analysis to compare groups and leave it to a presentation of various aspects. Given the heterogeneity of the products being compared, particularly the varying times until the expiry date or best-before date, we believe making generalized statements would be inappropriate.

In our study, we found that the pea protein products tested have higher numbers of bacterial genomic equivalents compared to the soya protein products (Addon 2). Overall, we had assumed that the information provided on the product labels would be somewhat associated with the bacterial counts detected in these products. We expected that products with higher bacterial counts would be more likely to display labels indicating the need for heat treatment, longer cooking times, or an expiry date rather than a best-before date. However, our finding did not support this assumption based on the tested products (Addon 3).

While the presence of lactic acid bacteria, which were the most prevalent bacteria detected, is not problematic, it is still important to investigate the reasons and specific criteria behind the decisions made for food labeling.

References: C. M. Liu, M. Aziz, S. Kachur, P.-R. Hsueh, Y.-T. Huang, P. Keim, L. B. Price, BactQuant: An enhanced broad-coverage bacterial quantitative real-time PCR assay, BMC Microbiology. 12:56 (2012). https://doi.org/10.1186/1471-2180-12-56.

R Core Team, R: A language and environment for statistical computing, (2021). https://www.R-project.org/.

H. Pagès, P. Aboyoun, R. Gentleman, S. DebRoy, (2021). Biostrings: Efficient manipulation of biological strings. R package version 2.62.0. https://bioconductor.org/packages/Biostrings

H. Wickham, (2016). ggplot2: Elegant Graphics for Data Analysis. Springer-Verlag New York. https://ggplot2.tidyverse.org

M. Ayrapetyan, J.D. Oliver, The viable but non-culturable state and its relevance in food safety, Current Opinion in Food Science. 8 (2016) 127-133. https://doi.org/10.1016/j.cofs.2016.04.010

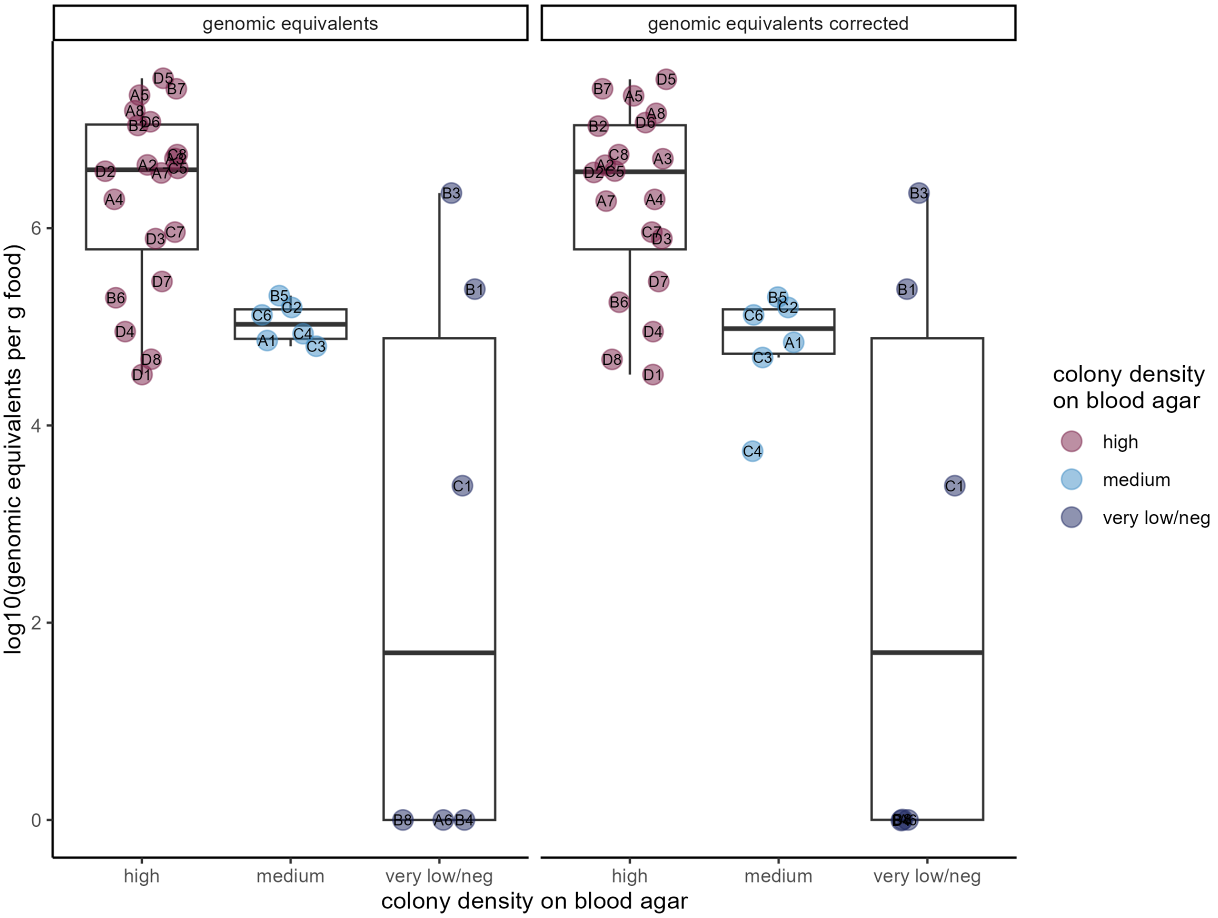

Addon 1 to Supplementary Notes 1: Genomic equivalents of each sample, grouped by the estimated growth density on Colombia blood agar at dilution 10-2. On the left side are the uncorrected genomic equivalents, on the right side the genomic equivalents corrected based on the 16S rRNA sequencing data.

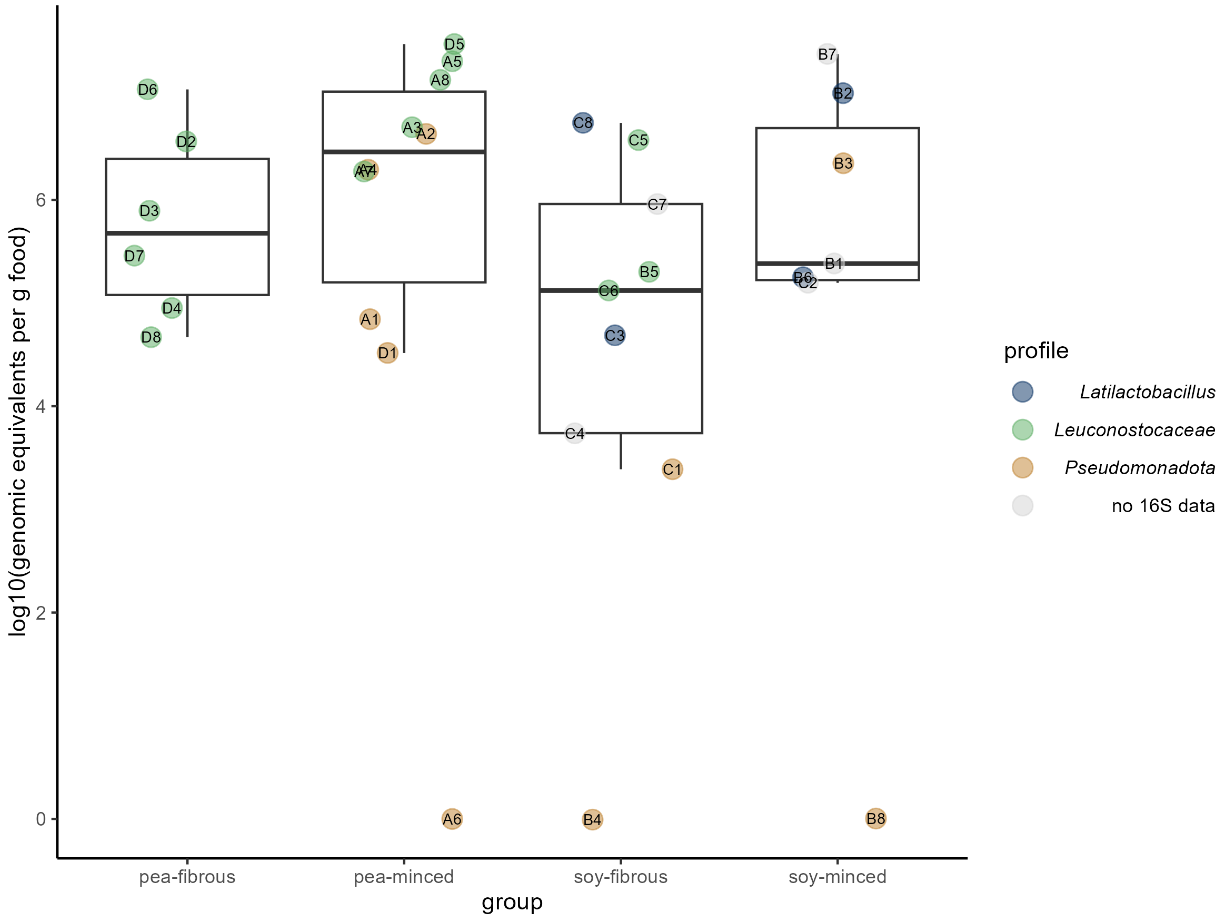

Addon 2 to Supplementary Notes 1: Genomic equivalents of each sample, grouped by the product group based on main protein source and texture. Points are filled based on the tSNE cluster profiles, described in the manuscript.

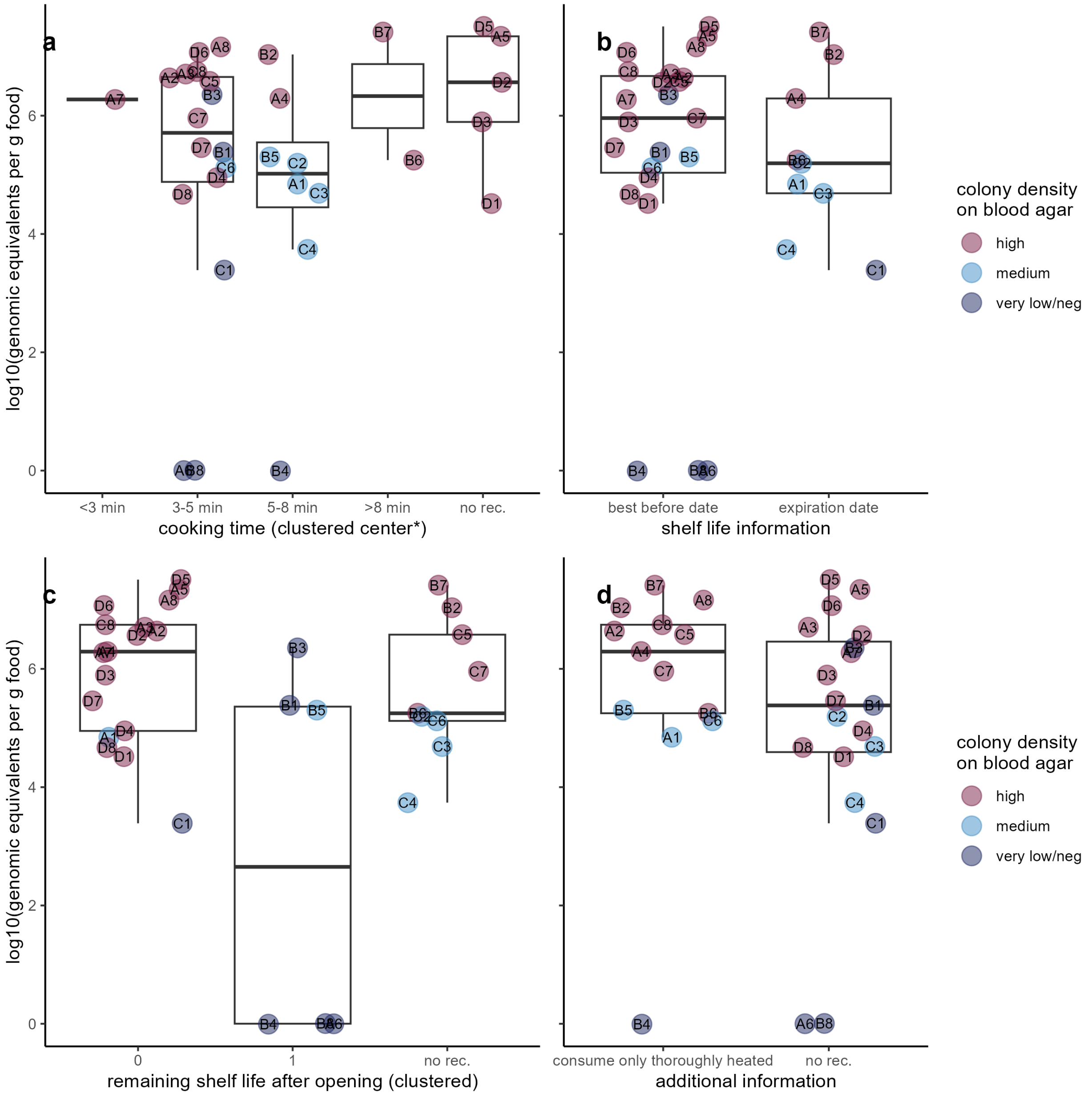

Addon 3 to Supplementary Notes 1: Genomic equivalents grouped by different types of product label information, a: comparison of the recommended cooking time (for data with a time range, the respective center of this range was used and assigned to a corresponding group), b: comparison between products with best before dates and with expiration dates, c: comparison of recommendation on the shelf life after opening the product (0 = within 1 day, 1 = within 2 or 3 days), d: comparison of products, that has the information "consume only thoroughly heated" with products without this information
